# Altered Maturation of Medullary TEC in EphB-Deficient Thymi Is Recovered by RANK Signaling Stimulation

**DOI:** 10.3389/fimmu.2018.01020

**Published:** 2018-05-09

**Authors:** Sara Montero-Herradón, Javier García-Ceca, Agustín G. Zapata

**Affiliations:** Department of Cell Biology, Faculty of Biology, Complutense University of Madrid, Madrid, Spain

**Keywords:** EphB, ephrin-B, medullary thymic epithelial cells, RANK, thymus development

## Abstract

In the present study, the relevance of EphB2 and EphB3 tyrosine kinase receptors for the maturation of medullary thymic epithelial cells (TECs) is analyzed. The absence of both molecules, but particularly that of EphB2, courses with altered maturation of medullary Cld3,4^hi^SSEA1^+^ epithelial progenitor cells, mature medulla epithelial cells, defined by the expression of specific cell markers, including UEA1, MHCII, CD40, CD80, and AIRE, and reduced expansion of medullary islets. *In vivo* assays demonstrate that these changes are a consequence of the absence of EphBs in both TECs and thymocytes. On the other hand, the changes, that remains in the adult thymus, correlated well with reduced proportions of E15.5 Vγ5^+^RANKL^+^ cells in EphB-deficient thymi that could result in decreased stimulation of RANK^+^ medullary TECs to mature, a fact that was confirmed by recovering of proportions of both CD40^hi^CD80^+^ and MHCII^hi^UEA1^+^ mature medullary TECs of mutant E14.5 alymphoid thymic lobes by agonist anti-RANK antibody treatment. Accordingly, the effects of EphB deficiency on medullary TECs maturation are recovered by RANK stimulation.

## Introduction

The thymus is a primary lymphoid organ morphological and functionally organized in two distinct histological compartments, the cortex and the medulla, whose origin and relationships remain obscure. Whereas in the thymic cortex thymocytes phenotypically mature and undergo positive selection of their TCR repertoire, in the medulla SP cells (both CD4^+^CD8^−^ and CD4^−^CD8^+^) are negatively selected to assure central tolerance and to impede self-reactive T-cell clones from escaping to periphery (1, 2).

The thymic primordium develops from the endoderm epithelium of the third pharyngeal pouch (3) which is later colonized by lymphoid progenitor (LP) cells coming from the fetal liver (4). Classically, differentiation of both cortical and medullary epithelium was explained according to a hierarchical model in which a common bipotent precursor cell was capable of giving rise to both cortical (c) thymic epithelial cells (TECs) and medullary (m) TECs (5). More recently, it has been demonstrated that several pools of thymic epithelial progenitor cells (TEPCs) would exist within the thymus (6): cells expressing cortical TEC markers are capable of producing both cTECs and mTECs (6–8), and Cld3,4^hi^SSEA1^+^ cells have self-renewing capacity and are capable of long-term production of mTECs, presumably constituting medullary committed TEPC (9). On the other hand, podoplanin^+^ mTEPCs of the cortico-medullary border have the potential to generate half of adult mTECs (10), and bipotent TEPCs exhibiting different phenotypes have been described in adult thymi (11, 12).

Nevertheless, relationships between these different progenitor cells and factors determining the cortical/medullary commitment remain elusive. It is generally assumed that thymocyte–TEC crosstalk regulates the maturation of both thymic cell components (13) and molecules of the TNF/TNFR superfamily seem to be important for medullary epithelium maturation (2, 14). The role of RANK/RANK ligand (RANKL) signaling appears central for the development of mTECs but other molecules of these families, such as osteoprotegerin (15) and LTβR (16) presumably modulate their effects and mTEC subsets are heterogeneous with respect to RANK expression (17). In turn, RANK signaling upregulates CD40 expression that also influences thymic medulla formation (18, 19).

Because, as mentioned above, mutual thymocyte–TEC influences play a major role in thymic epithelial maturation, we studied this process in a murine model in which thymocyte–TEC crosstalk is profoundly altered due to the lack of EphB receptors (20) that together with their ligands, ephrins-B, are involved in cell attachment–detachment and consequently in the organization of epithelial tissues (21).

In previous studies, we demonstrated that adult thymi of EphB2- and/or EphB3-deficient mice showed profound alterations in both cortical and medullary epithelium that appear during thymus ontogeny (22–24), and recently, we confirmed a major role of EphB3 in governing the development of cortical thymic epithelium (25). In the current study, we observed altered maturation of medullary epithelial cells, particularly evident in EphB2-deficient thymi, dependent on defects in both TECs and thymocytes that affected mTEPCs, including Cld3,4^hi^SSEA1^+^ cells, and mature mTECs, and reduced expansion of medullary islets. In correlation, mutant thymi contained significantly decreased proportions of E15.5 Vγ5^+^RANKL^+^ cells that presumably signaled less efficiently to RANK^+^ mTECs. In support of this, EphB-deficient E14.5 alymphoid thymic lobes stimulated with agonist anti-RANK antibodies recovered the wild type (WT) proportions of both CD40^hi^CD80^+^ and MHCII^hi^UEA1^+^ mature mTECs.

## Materials and Methods

### Mice

Both EphB2^−/−^ and EphB3^−/−^ mice generated in a CD1 background were kindly provided by Dr. Mark Henkemeyer (University of Texas, Southwestern Medical Center, Dallas, TX, USA). In all assays, WT and mutant CD1 mice were obtained from homozygous parents and, at least, five mice (WT and mutants) were used for each experimental group. All animals were bred and maintained under pathogen-free conditions in the animal care facilities of the Complutense University of Madrid. The day of vaginal plug detection was designated as day 0.5 for determining the age of fetuses.

### Animal Statement

The study was carried out in accordance with the recommendations of the “Ethic Committee for Animal Research” of Complutense University. The protocols were approved by the Regional Government of Madrid.

### Cell Suspensions and Flow Cytometry Analysis

Fetal TECs E14.5, E15.5 or E17.5 thymic lobes were isolated, disaggregated using trypsin 0.25× (Gibco, Invitrogen, Paisley, UK) and DNase I (0.1 mg/mL) (Roche, Germany) in RPMI 1640 for 20 min at 37°C and later gently pipetted to obtain a single-cell suspension. In the case of Claudin (Cld) 3,4 studies, tissue digestion was performed with Collagenase (1.2 mg/mL) (Roche, Germany). Grafted thymic lobes, seven day postnatal (PN), and adult thymi (6–8 weeks) were removed, cut with scissors and thymocytes were depleted by gently pipetting with a wide-bore glass pipette in cold RPMI 1640. Thymic fragments were poured twice, the supernatants mainly containing thymocytes were removed, and thymic fragments disaggregated by incubating with Liberase TM (Roche, Germany) at 1 U/mL together with DNAse I (0.1 mg/mL) for 15 min at 37°C in a water-bath. The cell suspensions obtained were washed and suspended in RPMI 1640 with 2% FBS and 10 mM ethylenediaminetetraacetic acid. Then, cell suspensions were stained for 15 min at 4°C in PBS 1% FBS with specific fluorescence conjugated mAb EpCAM-Alexa488 (clone G8.8), CD45-PE or PERCPCy5.5 or 647 (clone 30-F11), Ly51-PE (clone 6C3), MHCII-APC (clone M5/114.15.2), CD40-PE (clone 3/23), CD80-Alexa647 (clone 16-10A1), RANKL-PE (clone IK22/5) from BioLegend, CD45RB-PECy7 (clone C363.16A) and Vγ3TCR-FITC (Vγ5, clone 536) from BD Bioscience, and SSEA1-PerCP/Cy5.5 (clone 480) from Santa Cruz Biotechnology. For Claudin 3 and Claudin 4 (Thermo Fisher Scientific) or UEA1-Biotin (Ulex Europaeus Agglutinin Lectin 1) (Vector Laboratories) detection, after incubation with primary antibodies, cells were washed in PBS and then incubated with secondary antibody donkey anti-rabbit IgG-Alexa488 or Streptavidin-PECy7, respectively, for 15 min at 4°C in PBS 1% FBS. Before analysis, stained cell suspensions were washed in PBS, suspended in PBS 1% FBS, and analyzed in a FACSCalibur or FACSAriaIII devices (BD Biosciences) at the Flow Cytometry and Fluorescence Microscopy Center of the Complutense University of Madrid. In all cases, non-viable cells were excluded by forward-side scatter and the analyses were performed with FCS Express III software (*DeNovo* Software, Los Angeles, CA, USA).

### Fetal Thymus Organ Cultures (FTOCs) and RANK Signaling Activation

E14.5 thymic lobes isolated from both WT and EphB-deficient mice were cultured over 8 µm polycarbonate membranes (Merck Millipore, Germany) in RPMI 1640 (Lonza, Belgium) cell culture medium supplemented with 5% FBS, 1% penicillin and streptomycin, 1% glutamine, and 1% pyruvate for 6 days. Alymphoid FTOCs were obtained by supplying cell culture media with 1.35 mM of 2′-deoxyguanosine (2′-dGuo) (Sigma-Aldrich, St. Louis, MO, USA) for 6 days. The stimulation of RANK receptor was performed supplying alymphoid FTOCs with 10 µg/mL of an agonist anti-RANK antibody (26) (R&D Systems, USA) or anti-goat IgG, as isotype control (Jackson ImmunoResearch, PA, USA) for 4 days. After treatment, cell suspensions were obtained from lobes and analyzed by flow cytometry as described above.

### Grafting of Alymphoid Fetal Thymus Lobes Under the Kidney Capsule

E13.5 alymphoid thymus lobes isolated from both WT and EphB-deficient mice were obtained and cultured as previously described. Alymphoid thymus lobes from either WT or EphB-deficient mice were grafted under the kidney capsule of 2-month-old female WT or EphB-mutant mice. Briefly, the recipient mice were anesthetized with a ketamine–xylazine solution (ketamine: Ketolar 50 mg/mL, Pfizer Group, Spain, xylazine: Rompun 2%, Bayer, Germany) injected intraperitoneally. Kidney was exteriorized after dorsal incision; the connective capsule was separated from the renal parenchyma using a cannula and only one alymphoid lobe was implanted per kidney. Localization of the thymic lobe was visually secured. Finally, the muscle and skin were sutured with braided silk (Lorca Marín, Murcia, Spain). After 3 weeks, the animals were sacrificed and kidneys removed. Then, grafts were harvested and analyzed for cell content and development of TECs subsets by flow cytometry as previously described.

### Reaggregate Thymus Organ Cultures (RTOCs)

Wild type thymic cell suspensions obtained from E14.5 thymus lobes as previously described were incubated with either blocking anti-EphB2 or anti-EphB3 antibodies (2.5 μg/10^6^ cells) (R&D Systems, USA) or either anti-rat IgG2a (R&D Systems, USA) or anti-goat IgG isotype control (Jackson ImmunoResearch, PA, USA), respectively, for 1 h at 4°C. After incubation, cell suspensions were centrifuged for 5 min at 4°C, the pellets were reaggregated (RTOCs), transferred over 0.8 µm polycarbonate filters and cultured for 24 h in RPMI 1640 cell culture medium supplemented with 10% FBS, 1% penicillin and streptomycin, 1% glutamine, and 1% pyruvate, that contained either anti-EphB antibodies or isotype control antibodies. Then, RTOCs were included in Tissue-Tek OCT compound and frozen in liquid nitrogen for immunofluorescence analysis. Furthermore, RTOCs were also performed by using total thymic cells from either EphB2-, EphB3-deficient mice or WT cells, as control.

### Immunofluorescence and Semi-Quantification Analysis

6-µm thick thymic sections were obtained from E12.5–E15.5, E17.5, 7PN and adult WT and EphB-deficient mice or from RTOCs, fixed in acetone at room temperature for 10 min and air dried. Cryosections were stained with primary antibodies specific for either K5 (Covance, CA, USA), K8 (Developmental Studies Hybridoma Bank, Iowa City, IA, USA), AIRE (BD Bioscience, CA, USA), Claudin 3 and Claudin 4 (Thermo Fisher Scientific, USA), and MTS20 (Kindly gifted by Dr. Richard Boyd from Monash University) for 1 h at room temperature. After washing three times in cold PBS for 5 min, sections were incubated with the following secondary antibodies: donkey anti-rabbit IgG-AMCA, goat anti-rat IgM-Dylight594 (Jackson ImmunoResearch, PA, USA), donkey anti-rat IgG-Alexa594 or donkey anti-rabbit IgG-Alexa488 (Thermo Fisher Scientific, USA) for 45 min at room temperature. Sections were then washed in cold PBS three times for 5 min and mounted with antifade Prolong Gold (Thermo Fisher Scientific, USA). Samples were observed and photographed in a Zeiss Axioplan microscope provided with a Spot 2 digital camera at the Flow Cytometry and Fluorescence Microscopy Center (Complutense University, Madrid, Spain) equipped with Metamorph software (MDS Inc., Toronto, ON, Canada).

The proportions of Cld3,4^hi^ cells in both WT and EphB-mutant 7PN thymi were evaluated in pixels^2^ measuring the area occupied by Cld3,4^hi^ cells related to the total K8^+^ thymic area. A minimum of 10 non-overlapping serial sections from at least three independent experiments were evaluated. In WT and mutant RTOCs as well as in those supplied with anti-EphB antibodies, the size of K5^+^ medullary islets was determined in a minimum of five non-overlapping sections at different levels of, at least, three different RTOCs, obtaining the mean relative size of all medullary areas per section. Each area was counted as the pixels^2^ occupied by K5^+^ cells related to the total K8^+^ thymic area. These measures were performed blind to ensure the obtained results.

In both WT and mutant thymi, the number of AIRE^+^ cells was counted and related to the K5^+^ medullary area in pixels^2^ in a minimum of 10 non-overlapping medullary islets of at least three different thymi. Similarly, the numbers of K5^+^ medullary areas in WT and mutant RTOCs or in WT ones treated with anti-EphB antibodies were determined by counting the numbers of K5^+^ areas per section compared to the total K8^+^ thymic area expressed in pixels^2^ in a minimum of five non-overlapping sections of at least three different RTOCs.

All semi-quantitative analysis was carried out using Adobe Photoshop CS4 extended software (Adobe Systems Incorporated, San Jose, CA, USA).

### RNA Isolation, RT-PCR, and Real-Time PCR (qPCR)

Isolation of E15.5 and 7PN TECs (EpCAM^+^CD45^−^) from both WT and EphB-deficient thymi was performed in a FACSAriaIII cell sorter (BD Biosciences) at the Flow Cytometry and Fluorescence Microscopy Center of the Complutense University of Madrid. RNA from TEC suspensions was isolated using RNAqueous^®^-Micro Kit (Thermo Fisher Scientific, USA), according to the manufacturer’s instructions. The cDNA synthesis was performed by RT-PCR with the High-Capacity cDNA *Reverse* Transcription kit (Thermo Fisher Scientific, USA), using 0.1 µg of RNA according to the manufacturer’s instructions. The expression of different genes was determined by real-time PCR (qPCR) using Power SYBR Green PCR Master Mix (Thermo Fisher Scientific, USA) together with specific primers for HPRT1 (*Forward*: cctcctcagaccgcttttt; *Reverse*: aacctggttcatcatcgctaa); CCL19 (*Forward*: tgtggcctgcctcagattat, *Reverse*: agtcttccgcatcattagcac), LTβR (*Forward*: gctccaggtacctcctactcg, *Reverse*: atggccagcagtagcattg), or RANK (*Forward*: gtgctgctcgttccactg, *Reverse*: agatgctcataatgcctctcct). Primer sequences were identified using the Universal Probe Library Assay Design Center application (Roche) and produced by Sigma Aldrich (St. Louis, MO, USA). Efficiency of amplification reaction and Ct values for each gene were obtained using a 7900HT Fast Real-Time PCR system with SDS2.3 software at the Genomic Center (Complutense University, Madrid, Spain). The relative expression of each sample was normalized to HPRT1 values and represented as RQ (2^−ΔΔCt^). Data show the media of the three independent experiments.

### Statistical Analysis

The results were expressed as mean ± SD corresponding to, at least five independent experiments for phenotypical analysis. The significance of differences with respect to control values was analyzed by the Student’s *t-test* after analysis of *f-test* data. In the case of gene expression analyses, the relative expression of mutant values of the three independent experiments was compared to the WT one using the one-*t test*. The used software for statistical procedures consists of Microsoft Excel 2010 (Redmond, MA, USA) and GraphPad Prism 5 (La Jolla, CA, USA). The significance probability between WT and mutant values is indicated as *p ≤ 0.05; **p ≤ 0.01; ***p ≤ 0.005, whereas differences between mutant values is expressed as ^#^*p* ≤ 0.05; ^##^*p* ≤ 0.01; ^###^*p* ≤ 0.005.

## Results

### Altered Maturation of UEA1^+^ mTECs Cells in EphB-Deficient Thymi

**Figure 1:**
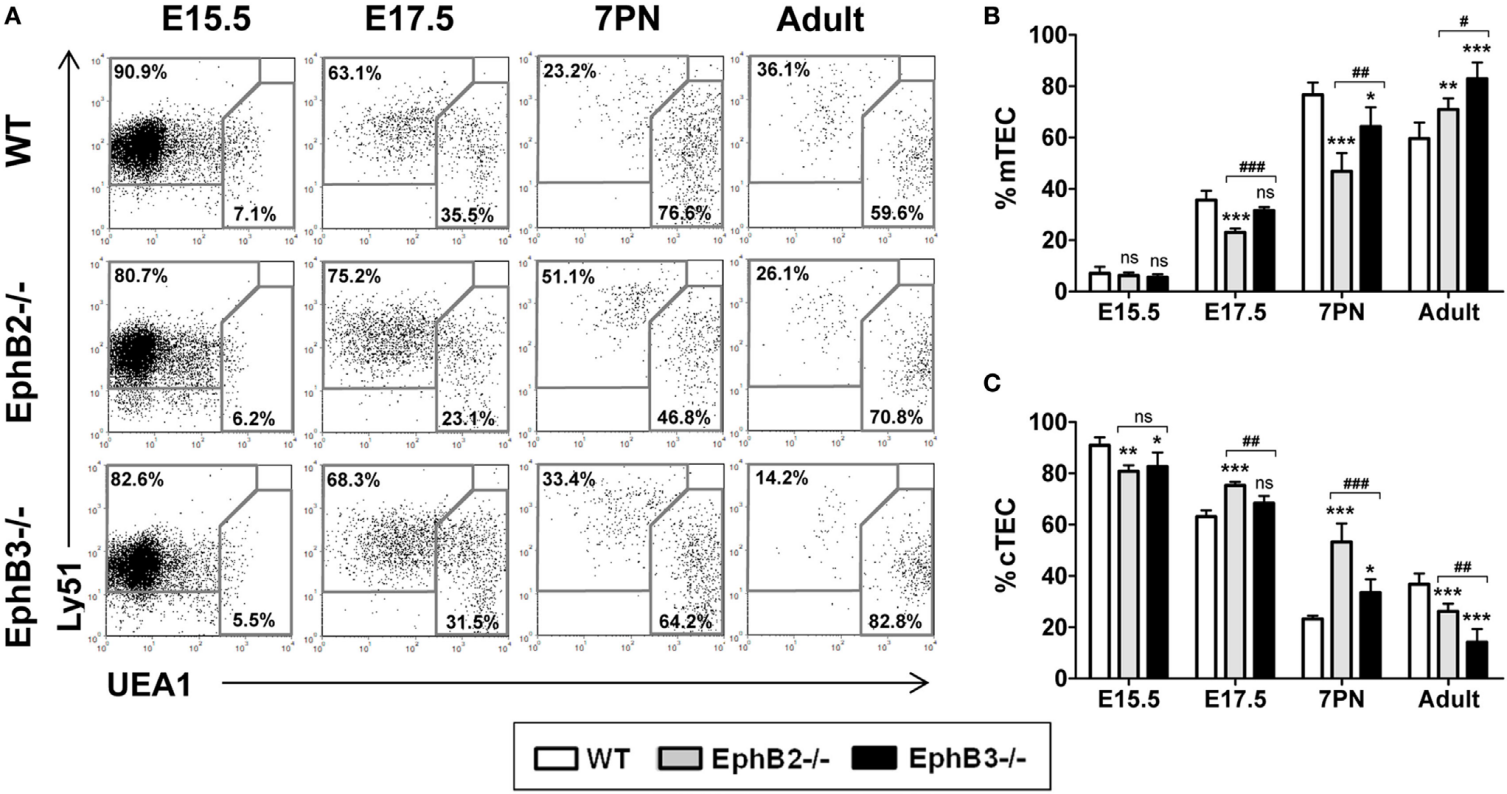
Thymic epithelial cell (TEC) subsets defined according to Ly51 and UEA1 expression in fetal (E15.5, E17.5), postnatal (7PN), and adult wild type (WT) and EphB-deficient mice. **(A)** *Dot plots* representative of at least five analyses, gating in total WT and mutant EpCAM^+^CD45^−^ epithelial cells, show the maturation of both medullary TECs (mTECs) (Ly51^−^UEA1^+^) and cTECs (Ly51^+^UEA1^−^). Numerical values indicate the frequency of each cell population. **(B,C)** Proportion of mTEC **(B)** and cTEC **(C)** subsets at different stages of thymus development. In both figures, the significance of the Student’s *t*-test probability between WT and mutant values is indicated: *p ≤ 0.05; **p ≤ 0.01; ***p ≤ 0.005; or when mutant values are compared between them: ^#^*p* ≤ 0.05; ^##^*p* ≤ 0.01; ^###^*p* ≤ 0.005; ns, non-significant.

We first evaluated the development of TECs by analyzing the expression of a cTEC marker, Ly51, and another medullary one, UEA1, in the total EpCAM^+^CD45^−^ TECs in both WT and EphB-deficient thymi at the last fetal stages (E15.5, E17.5), 7 days after birth (7PN) when the medulla begins its expansion (27), and in adults (6–8 weeks). In both WT and EphB-mutant thymi, there was a gradual upregulation of UEA1 expression (Figure 1A). At E15.5, in both WT and EphB-mutant thymi the main TEC population corresponded to Ly51^+^UEA1^−^ cTECs with some UEA1-expressing cells. These cTECs reached the lowest values at 7PN (Figure 1A). By contrast, there was a gradual increase in the proportion of Ly51^−^UEA1^+^ mTECs that represented the most TECs at 7PN (Figure 1A), as also reported by other authors (28).

No differences in the mTEC proportions were observed at E15.5 between EphB-deficient thymi and WT ones (Figure 1B). At both E17.5 and 7PN, the proportions of Ly51^−^UEA1^+^ mTEC populations were significantly lower in EphB2^−/−^ thymi, but only in EphB3^−/−^ ones at 7PN as compared to WT values (Figure 1B). In correlation, as compared with WT values, the proportions of mutant cTECs (Figure 1C) were significantly lower at E15.5 but higher later, at E17.5 and 7PN, with significant differences in EphB2^−/−^ thymi. In adult thymi, the proportions of Ly51^−^UEA1^+^ cells (Figure 1B) were significantly higher in both mutant mice in close correlation with decreased proportions, particularly in EphB3-deficient thymi, of cTECs (Figure 1C). All these data demonstrated a more severe phenotype according to Ly51/UEA1 expression in the EphB2^−/−^ thymi than in the EphB3^−/−^ ones.

### Maturation of TEC Populations Defined by the Expression of Medullary Functional Epithelial Markers (MHCII, CD80, AIRE) Confirmed the Altered Maturation of mTECs in EphB-Deficient Thymi

**Figure 2:**
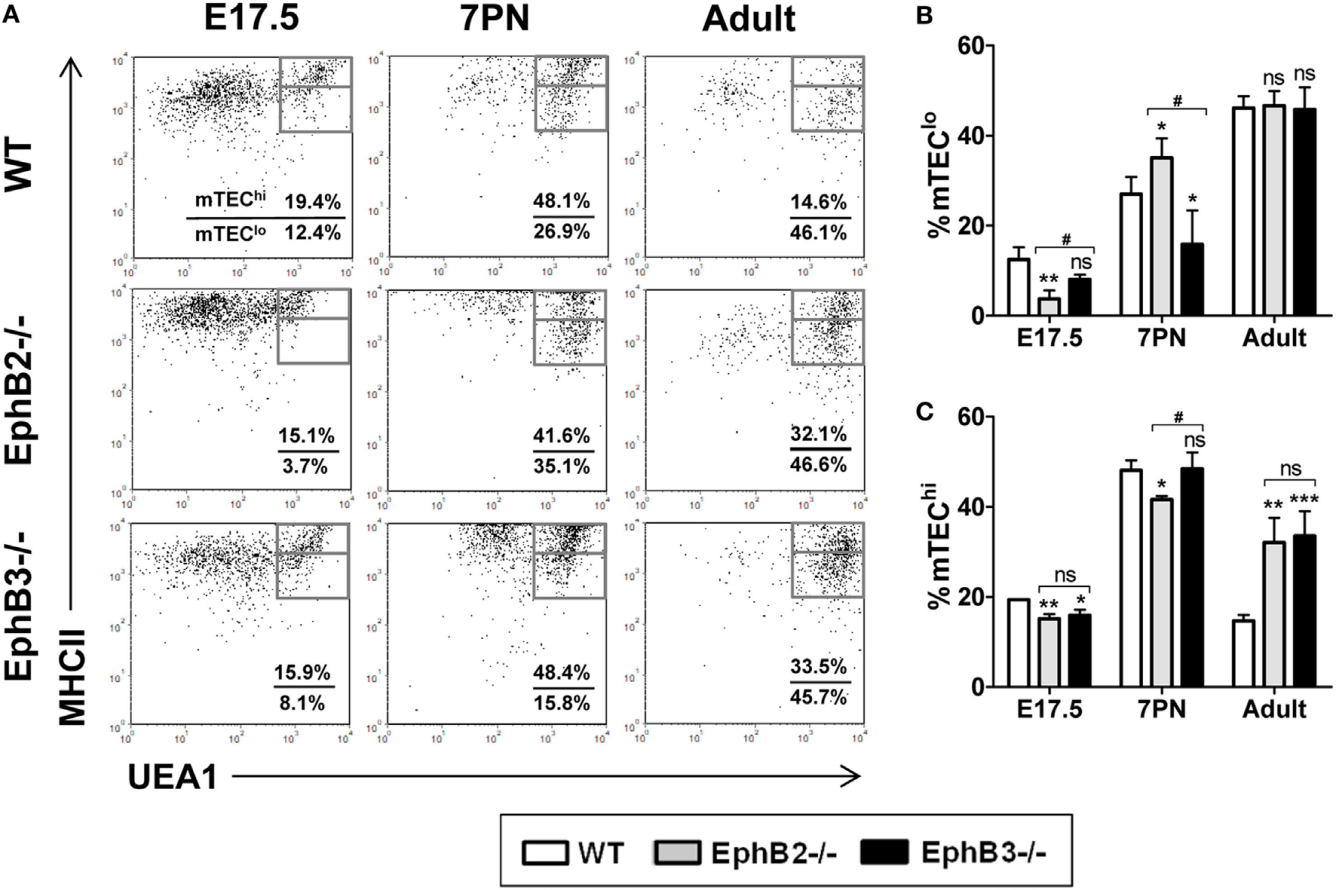
Medullary TECs (mTEC) subsets defined by the expression of UEA1/MHCII cell markers in E17.5, 7PN, and adult thymi in both wild type (WT) and EphB-deficient mice. **(A)** *Dot plots* show mTEC^lo^ (MHCII^med^UEA1^+^) and mTEC^hi^ (MHCII^hi^UEA1^+^) cell subpopulations according to MHCII and UEA1 expression, gated in total EpCAM^+^CD45^−^ cells. Numerical values show the frequency of each population. **(B,C)** Figures show the proportions of mTEC^lo^ **(B)** and mTEC^hi^ cells **(C)**. The significance of the Student’s *t*-test probability is indicated: *p ≤ 0.05; **p ≤ 0.01; ***p ≤ 0.005; and ^#^*p* ≤ 0.05 between mutants; ns, non-significant.

During development, MHCII expression, which is involved in intrathymic thymocyte selection and maturation of medullary epithelium (29), was upregulated first in the UEA1^−^ cortical epithelium and later in the UEA1^+^ mTECs (Figure 2A). In WT thymi, the proportions of both MHCII^med^UEA1^+^ mTECs^lo^ (Figure 2B) and MHCII^hi^UEA1^+^ mTECs^hi^ (Figure 2C) increased between E17.5 and 7PN. The pattern of evolution was similar in mutant thymi but with significantly different values, particularly in the case of EphB2^−/−^ thymi that once again showed a more severe phenotype than that of EphB3^−/−^ thymi.

At E17.5, the values found in EphB2^−/−^ thymi in both mTEC subsets were significantly lower than in WT ones (Figures 2B,C). At that stage, the proportions of EphB3^−/−^ UEA1^+^ cells were only significantly lower in the most mature MHCII^hi^UEA1^+^ cell population (Figure 2C), as compared to WT values. At 7PN, as compared to WT values, the proportions of mTEC^lo^ were significantly higher in EphB2^−/−^ thymi but lower in EphB3^−/−^ ones (Figure 2B), whereas the percentage of EphB2^−/−^ mTEC^hi^ showed lower values than WT, without differences between WT and EphB3^−/−^ thymi (Figure 2C). When the proportions of distinct cell subsets were compared between mutant thymi, differences appeared in the proportions of E17.5 mTEC^lo^ (Figure 2B) and in both mTEC^lo^ and mTEC^hi^ at 7PN (Figures 2B,C). No differences occurred in the proportions of mTEC^lo^ of WT and mutant adult thymi (Figure 2B), whereas those of the most mature mTEC had significantly increased in mutant ones with respect to WT values (Figure 2C).

**Figure 3:**
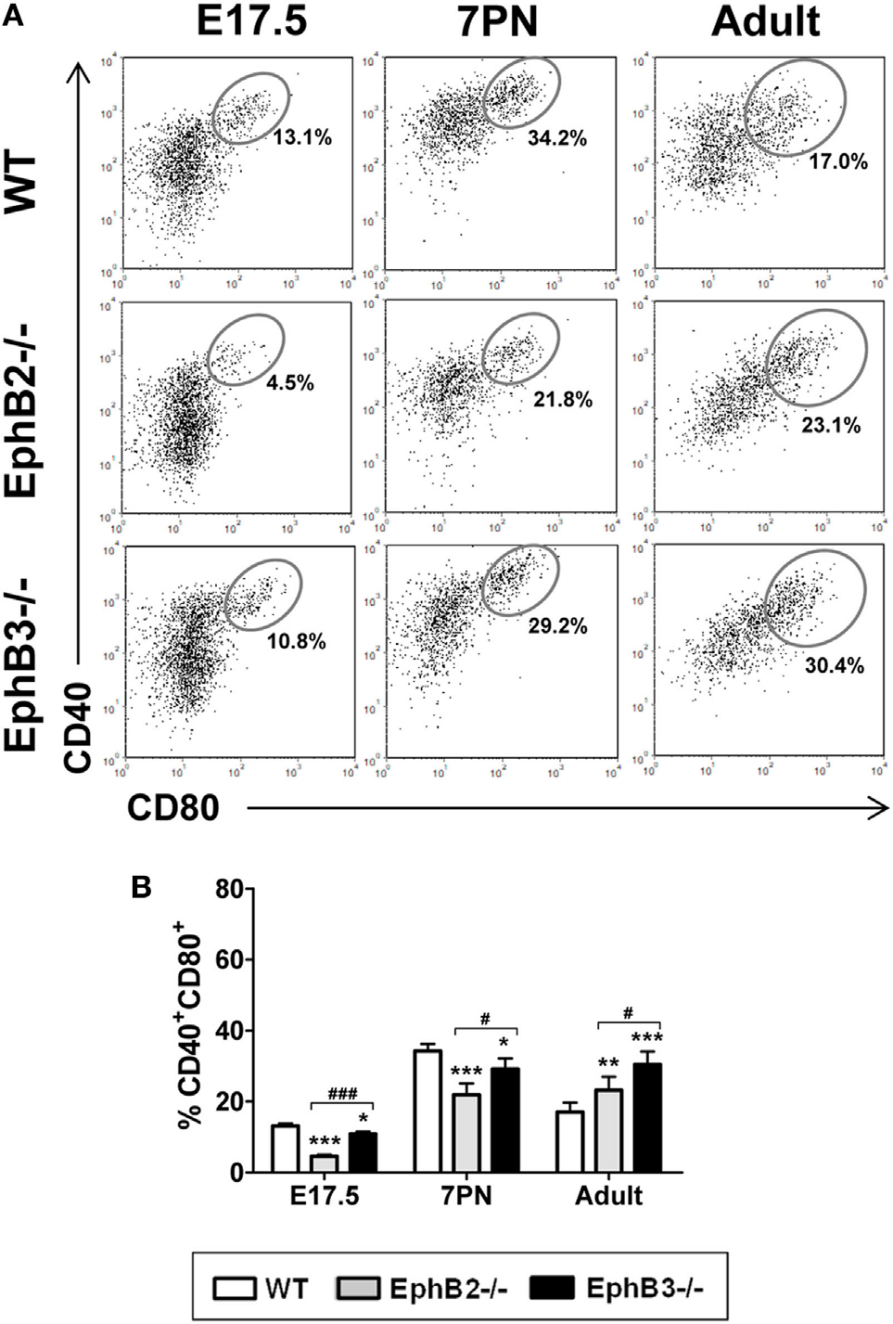
Proportions of the CD40^+^CD80^+^ cells in developing wild type (WT) and EphB-deficient thymi. **(A)** *Dot plots* show the evolution of CD40^+^CD80^+^ cells along thymus development. Indicated values show the frequency of distinct cell populations. **(B)** The comparative analysis of CD40^+^CD80^+^ cells shows reduced proportions in mutant thymi, more evident in the absence of EphB2, at E17.5 and 7PN and increased values in the adult thymi. The significance of the Student’s *t*-test probability is indicated: *p ≤ 0.05; **p ≤ 0.01; ***p ≤ 0.005; and ^#^*p* ≤ 0.05; ^###^*p* ≤ 0.005 between mutants; ns, non-significant.

Differential expression of CD40/CD80 co-stimulatory molecules (Figure 3A) also allowed mTEC maturation to be studied (30). At E17.5, the proportions of CD40^+^CD80^+^ mTECs were still low, particularly in mutant thymi (Figure 3B). Even values in EphB2^−/−^ thymi were significantly lower than those observed in both WT and EphB3^−/−^ thymi (Figure 3B). At 7PN, the values increased in both control and mutants but the latter exhibited significantly lower proportions (Figure 3B). Again, in adult thymi, the proportions of CD40^+^CD80^+^ mTECs were significantly higher in mutant thymi than in WT ones (Figure 3B).

**Figure 4:**
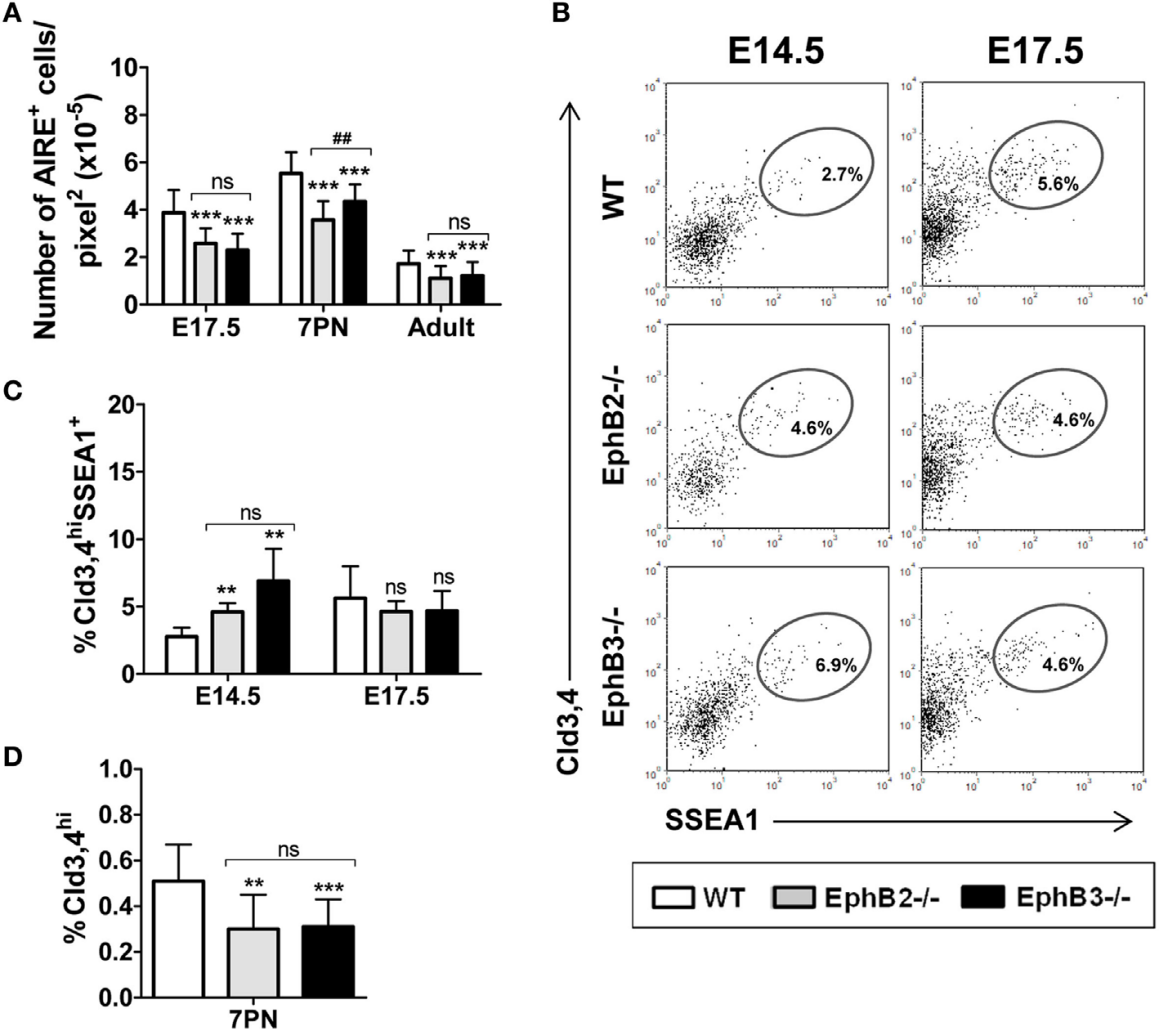
Presence of AIRE^+^ cells and Cld3,4^+^ cells in fetal, postnatal, and adult wild type (WT) and EphB-deficient thymi. **(A)** Semi-quantitative analysis of AIRE^+^ cells reveals that mutant thymi have significantly lower numbers of these cells at all studied stages. **(B)** *Dot plots* show the expression of Cld3,4^hi^SSEA1^+^ medullary TECs progenitor cells in both WT and EphB-mutant thymi at E14.5 and E17.5, indicating their frequencies. **(C)** Comparative analysis of the proportions of Cld3,4^hi^SSEA1^+^ cells in WT and EphB-deficient thymi at E14.5 and E17.5. **(D)** Semi-quantitative analysis of the Cld3,4^hi^ expression in WT and mutant thymi at 7PN. The significance of the Student’s *t*-test probability is indicated: **p ≤ 0.01; ***p ≤ 0.005; and ^##^*p* ≤ 0.01 between mutants; ns, non-significant.

We also studied possible changes in the numbers of AIRE^+^ cells that play a key role in the functional maturation of thymic medulla (31). Our semi-quantitative study showed that the numbers of AIRE^+^ cells that occurred in the K5^+^ thymic medullary region (Figure S1 in Supplementary Material) reached the highest values in both WT and mutant 7PN thymi dropping down later (Figure 4A). In all studied stages, the proportions of AIRE^+^ cells were significantly lower in both mutant thymi than in the WT ones, with significant differences between 7PN EphB2^−/−^ and EphB3^−/−^ thymi (Figure 4A).

### Altered Maturation of Different Claudin 3,4^+^ Progenitor Cells in Fetal and Postnatal Eph-Deficient Thymi

The above-described results pointed to an altered maturation of distinct mTEC subsets identified by different specific cell markers in the absence of EphB receptors. We, then, evaluated whether this finding was related to altered numbers of medullary progenitor cells. Although results of TEC lineages and the nature of medullary progenitor cells remain controversial, Cld3,4-expressing epithelial cells that occur in the lumen of early thymic primordium have been suggested to be committed precursor cells of the medullary epithelial cell lineage (32) and more recently, a subset of this cell population, Cld3,4^hi^SSEA1^+^ cells, was reported to show self-renewal potential and capabilities for long term generation of mTECs (9). On the other hand, MTS20/24 antibodies were used to characterize immature epithelial cells, originally described as TEPCs (33) but later discarded (34).

The histological analysis of E12.5 and E13.5 WT and mutant thymic sections showed MTS20^+^ cells distributed throughout the thymic parenchyma, although in some central areas they co-localized with Cld3,4^hi^ cells (Figure S2A in Supplementary Material, insets). From E13.5 onward, the Cld3,4^hi^MTS20^hi^ cells decreased until they practically disappeared at E15.5 (Figure S2A in Supplementary Material), as previously described by other authors (32). However, in mutant thymi this cell subset, although decreased, remained still evident at that developmental stage, reflecting its slow maturation compared to the condition of WT thymi (Figure S2A in Supplementary Material).

The maturation of Cld3,4^hi^SSEA1^+^ cells was analyzed at E14.5 and E17.5 (Figure 4B) because this cell population postnatally decreased (9). The proportions of Cld3,4^hi^SSEA1^+^ cells in mutant thymi accumulated at E14.5 and reached control values at E17.5, when they increased in the WT thymi (Figure 4C), again supporting a slow maturation.

Although the activity of Cld3,4^hi^SSEA1^+^ cells significantly declines in postnatal thymi (9), increased proportions of Cld3,4^hi^ cells (32) have been reported in correlation with postnatal medulla expansion. We, therefore, histologically analyzed the presence of Cld3,4^hi^ cells (green) in the K8^+^ thymic parenchyma (blue) of both 7PN WT and mutants (Figure S2B in Supplementary Material). The semi-quantitative study revealed that the expression of Cld3,4^hi^ cells in EphB2^−/−^ and EphB3^−/−^ thymi was significantly lower than in WT ones (Figure 4D). These results confirmed that absence of EphB2 or EphB3 also coursed with altered maturation of Cld3,4^hi^ cells in postnatal thymi.

### Abnormal Development of Medullary Islets Observed in EphB-Deficient Thymi Can Be Reproduced in RTOCs-Treated With Either Blocking Anti-EphB2 or Anti-EphB3 Antibodies

**Figure 5:**
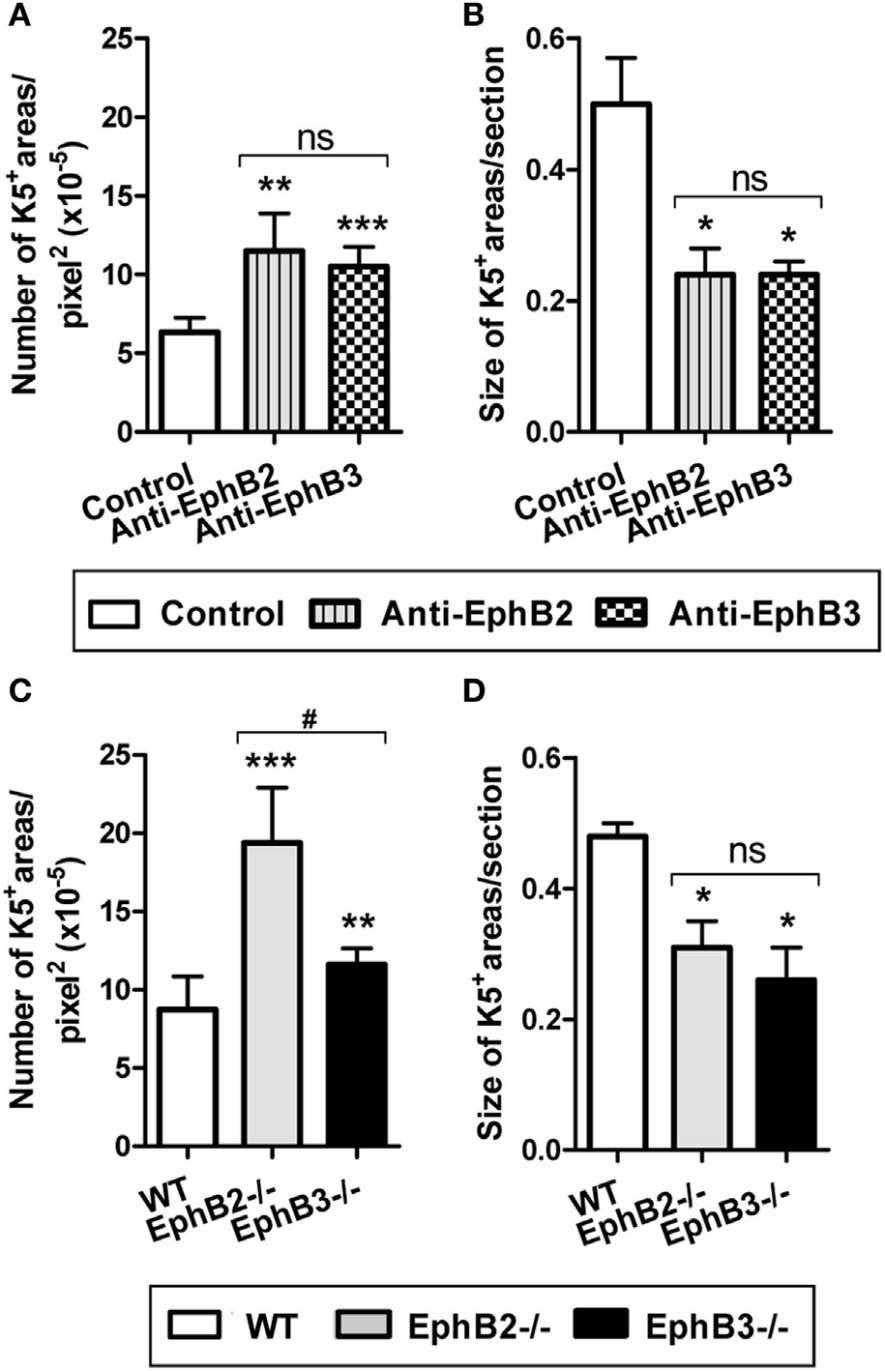
Number and size of K5^+^ medullary areas in either wild type (WT) reaggregate thymus organ cultures (RTOCs) treated with either anti-EphB2 or anti-EphB3 antibodies or established with EphB-deficient cells. Semi-quantitative analysis of the number **(A)** and size **(B)** of K5^+^ areas in WT RTOCs treated with anti-EphB2 or anti-EphB3 or isotype control (control) antibodies. Number **(C)** and size **(D)** of K5^+^ areas in RTOCs established either with EphB2^−/−^ or EphB3^−/−^ or WT cells. A minimum of five non-overlapping sections of at least three different RTOCs was analyzed. The significance of the Student’s *t*-test probability is indicated: *p ≤ 0.05; **p ≤ 0.01; ***p ≤ 0.005; and ^#^*p* ≤ 0.05 between mutants; ns, non-significant.

Mature thymic medulla is formed from individual islets that, after birth, fuse constituting a single central medulla surrounded by some islets more or less connected to each other (35). However, in developing EphB-deficient thymi, particularly in EphB2^−/−^ ones, the organization of a single, central medulla seems to be impaired and small, isolated foci arranged throughout the organ remain (22). In order to confirm that this medullary phenotype was related to the lack of EphB signaling, we evaluated whether the *in vitro* blocking of EphB signaling in E14.5 WT thymic reaggregates (RTOCs) by treatment with either blocking anti-EphB2 or anti-EphB3 antibodies mimicking the medullary condition of developing EphB-deficient thymi. The treated RTOCs showed smaller and more numerous K5^+^ areas scattered throughout the K8^+^ thymic parenchyma as compared with control ones (Figure S3 in Supplementary Material). Similar results were obtained when RTOCs were performed using WT, EphB2- or EphB3-deficient thymic-derived cells (Figure S3 in Supplementary Material). Semi-quantitative analysis of the size and numbers of K5^+^ medullary areas of both WT and EphB-deficient RTOCs confirmed the reported morphological differences. In addition, the numbers of K5^+^ areas by RTOC section were significantly higher in both treated RTOCs and EphB-deficient RTOCs than in control RTOCs and WT ones, respectively (Figures 5A,C). In particular, numerous K5^+^ areas occurred in EphB2-deficient RTOCs (Figure 5C). On the other hand, the mean size of K5^+^ areas was significantly lower in treated and mutant RTOCs than in the respective control ones (Figures 5B,D).

### Comparative Analysis of the *In Vivo* Maturation of WT or EphB-Deficient Alymphoid FTOCs Under the Kidney Capsule of Mutant or WT Mice, Respectively

**Figure 6:**
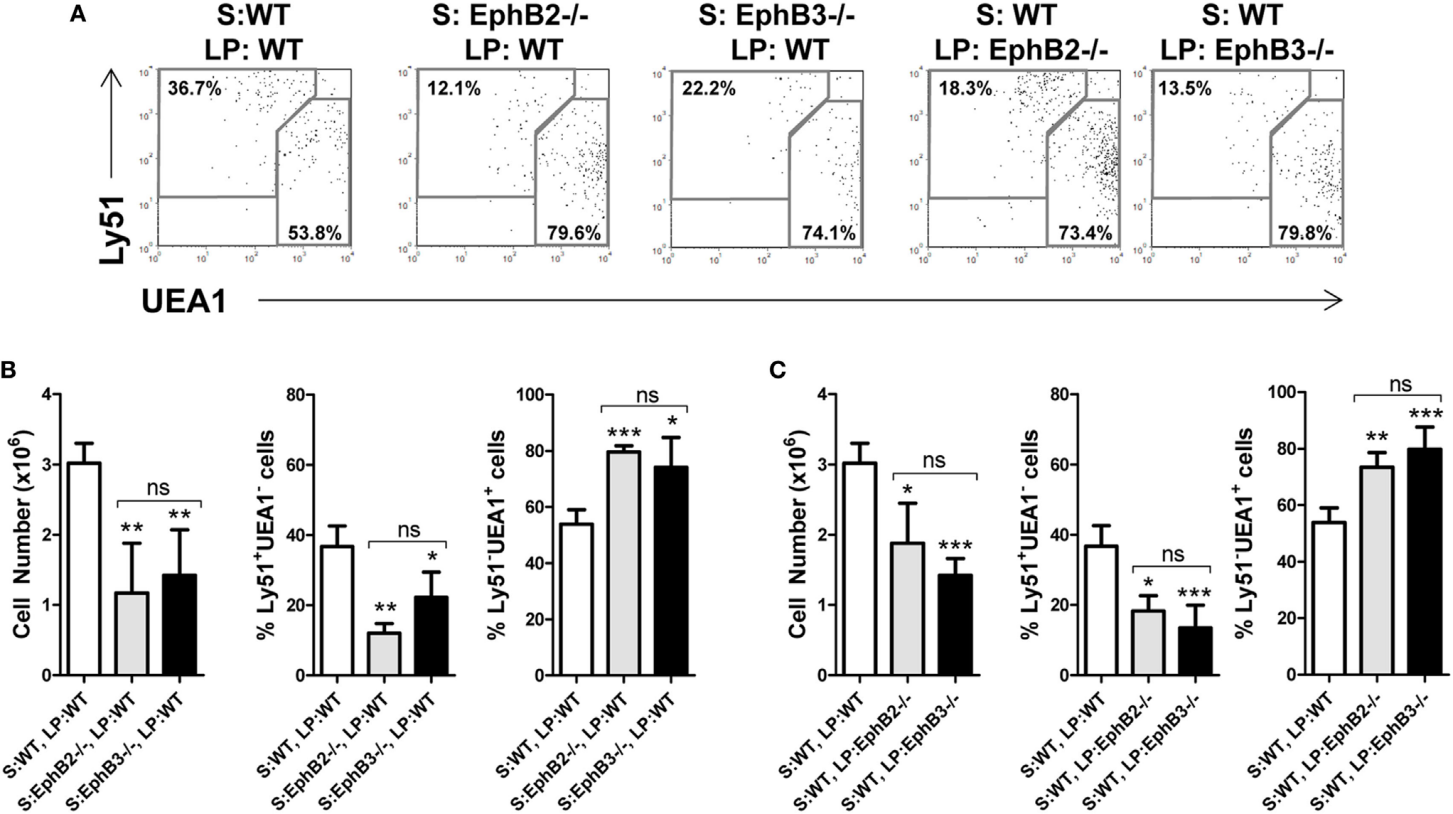
Thymic epithelial cell (TEC) maturation in alymphoid wild type (WT) or EphB^−/−^ fetal thymus organ cultures (FTOCs) grafted under the kidney capsule of WT or EphB^−/−^ mice. Alymphoid WT or EphB^−/−^ FTOCs (S) were colonized by either WT or EphB^−/−^ host lymphoid progenitors (LPs) during 3 weeks. **(A)** *Dot plots*, gated in total EpCAM^+^CD45^−^, show the proportions of cTECs (Ly51^+^UEA1^−^) and medullary TECs (mTECs) (Ly51^−^UEA1^+^) in different stroma and LP combinations (S: WT, LP: WT; S: EphB2^−/−^, LP: WT; S: EphB3^−/−^, LP: WT; S: WT, LP: EphB2^−/−^; and S: WT, LP: EphB3^−/−^). **(B)** Significantly lower cellularity and cTEC proportions, but higher mTEC percentages in EphB2- or EphB3-deficient stroma seeded by WT LPs as compared to control conditions (S: WT, LP: WT). **(C)** Similar results are observed when WT stroma is grafted under the kidney capsule of EphB2- or EphB3-deficient mice. The significance of the Student’s *t*-test probability is indicated: *p ≤ 0.05; **p ≤ 0.01; ***p ≤ 0.005; ns, non-significant.

We evaluated whether the altered mTEC development observed in EphB-mutant thymi was due exclusively to the absence of either EphB2 or EphB3 on TECs, or whether thymocytes also contributed non-autonomously. For this purpose, we grafted E13.5 WT alymphoid FTOCs under the kidney capsule of mutant host mice or mutant alymphoid FTOCs in WT mice comparatively analyzing the maturation of TEC population defined by Ly51/UEA1 expression 3 weeks later (Figure 6A). At this time, as compared with control conditions, WT thymic lobes grafted into WT mice, both EphB2^−/−^ and EphB3^−/−^ FTOCs grafted into WT host mice showed significantly reduced cellularity and increased proportions of mTECs that correlated with reduced cTECs (Figure 6B). In these experimental conditions, there were no differences between the two mutant FTOCs grafted (Figure 6B). On the other hand, when WT alymphoid FTOCs were colonized by EphB2^−/−^ or EphB3^−/−^ LPs (Figure 6C), the yielded lobes also showed significant reduced cell numbers, increased proportions of mTECs, and lower values of cTECs, as compared to those seeded by WT lymphoid cells (Figure 6C).

### Changes in the Expression of Molecules Known to Be Involved in mTEC Maturation in Eph-Deficient Thymi

**Figure 7:**
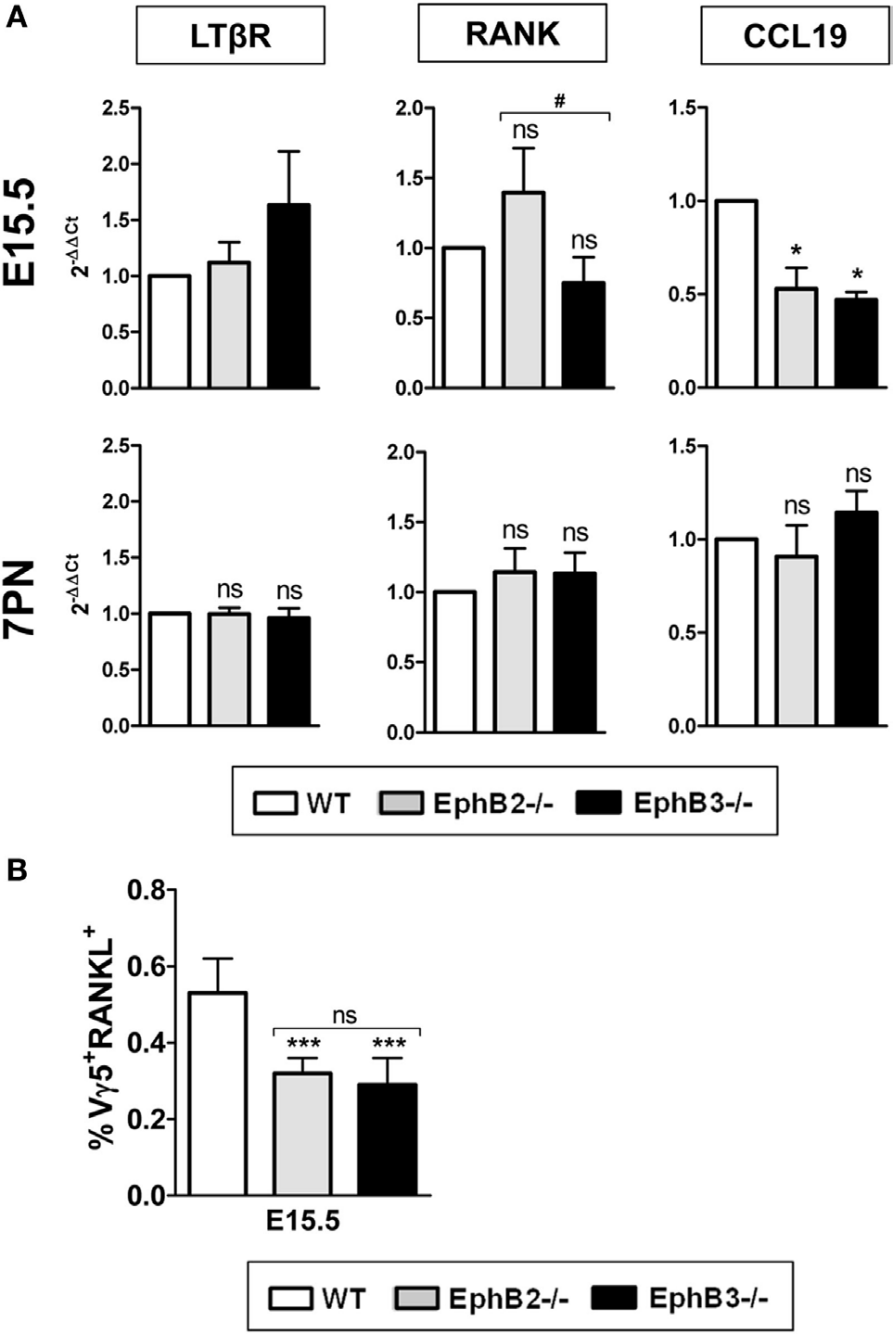
Expression of different molecules involved in medullary TEC development and presence of Vγ5^+^RANKL^+^ thymocytes in fetal wild type (WT) and EphB-deficient thymi. Relative expression of LTβR, RANK, and CCL19 transcripts in mutant thymi respect to WT ones **(A)**. Proportions of CD45RB^+^Vγ5^+^RANKL^+^ (Vγ5^+^RANKL^+^) thymocytes in both E15.5 WT and mutant thymi **(B)**. The significance of the Student’s *t*-test probability is indicated: *p ≤ 0.05; ***p ≤ 0.005; and ^#^*p* ≤ 0.05; ns, non-significant.

In order to clarify the altered maturation of EphB-deficient mTEC, we studied possible changes in the expression of some molecules of the TNFR superfamily (LTβR and RANK) and chemokines (CCL19) known to be involved in medulla epithelium development. Our study began at E15.5 when the first mTECs appeared in the developing thymus and later, at 7PN, when the thymic medulla expanded. No changes were found in the transcripts of either LTβR or RANK from EphB-deficient thymi at E15.5 and 7PN (Figure 7A), whereas CCL19 transcripts at E15.5, but not at 7PN, were significantly lower in mutant thymic than in WT ones (Figure 7A).

### The Proportions of Vγ5^+^RANKL^+^ Cells Were Significantly Reduced in Fetal EphB-Deficient Thymi

In the adult thymus, the activation of RANK signaling in TECs is mediated by RANKL expressed on positively selected SP CD4^+^ thymocytes (36), while in the fetal thymus, where there are still no SP thymocytes, both lymphoid tissue inducer cells (26) and canonical Vγ5^+^ T cells express RANKL and promote maturation of immature CD80^−^AIRE^−^ to mature CD80^+^AIRE^+^ TECs (37). As shown in Figure 7B, the proportions of CD45RB^+^Vγ5^+^RANKL^+^ (Vγ5^+^RANKL^+^) cells were very low in both WT and mutant thymi although significantly lower in the latter.

### Activation of RANK/RANKL Signaling in EphB-Deficient mTECs Recovers Altered mTEC Maturation

**Figure 8:**
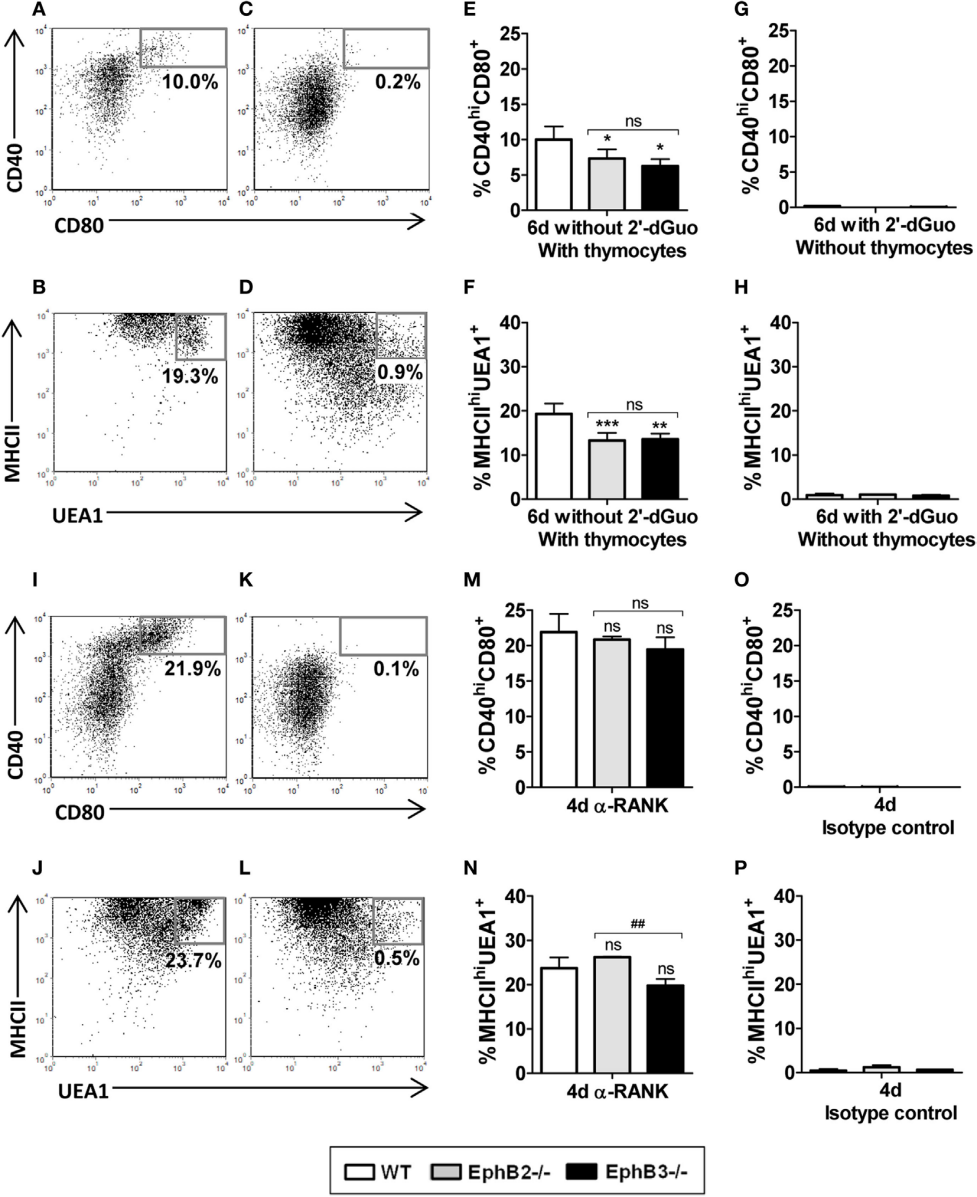
Analysis of the proportions of CD40^hi^CD80^+^ and MHCII^hi^UEA1^+^ medullary TECs (mTECs) in wild type (WT) and mutant fetal thymus organ cultures (FTOCs) cultured in presence or absence of thymocytes and after anti-RANK stimulation. **(A,B)** *Dot plots*, gated in total EpCAM^+^CD45^−^, show the presence of CD40^hi^CD80^+^ **(A)** and MHCII^hi^UEA1^+^ **(B)** mTECs subsets after 6 days of culture in the presence of thymocytes (without 2′-dGuo) but not in their absence (with 2′-dGuo) [**(C,D)**, respectively]. **(E–H)** Proportions of CD40^hi^CD80^+^ **(E)** and MHCII^hi^UEA1^+^ mTECs **(F)** in either EphB-deficient or WT FTOCs in the presence of thymocytes or in absence of thymocytes [**(G,H)**, respectively]. **(I,J)** *Dot plots* showing proportions of both CD40^hi^CD80^+^ **(I)** and MHCII^hi^UEA1^+^ **(J)** mTECs in alymphoid FTOCs after 4 days of anti-RANK treatment or its absence [**(K,L)**, respectively]. Note the lack of differences in the proportions of CD40^hi^CD80^+^ **(M)** and MHCII^hi^UEA1^+^ mTECs **(N)** between alymphoid mutants and WT FTOCs. **(O,P)** Proportions of both CD40^hi^CD80^+^ **(O)** and MHCII^hi^UEA1^+^ **(P)** mTECs in alymphoid FTOCs after 4 days in the presence of isotype control. Indicated numerical values in *dot plots* show the frequency of each cell population. The significance of the Student’s *t*-test probability is indicated: *p ≤ 0.05; **p ≤ 0.01; ***p ≤ 0.005; and ^##^*p* ≤ 0.01 between mutants; ns, non-significant.

On the basis of these results, we speculated that RANK/RANKL signaling could be altered in mutant thymi as a consequence of defective thymocyte-TEC crosstalk demonstrated in the absence of Eph and/or ephrins B (20), affecting proper mTEC development. In order to confirm this hypothesis, we first analyzed *in vitro* mTEC maturation in the presence or absence of thymocytes. In the presence of thymocytes (FTOCs without 2′-dGuo) (Figures 8A,B), but not in their absence (alymphoid cultures treated with 2′-dGuo) (Figures 8C,D), both WT and mutant FTOCs contained both CD40^hi^CD80^+^ cells (Figure 8A) and MHCII^hi^UEA1^+^ cells (Figure 8B). Nevertheless, mutant FTOCs showed reduced proportions of the two cell types of mTECs, CD40^hi^CD80^+^ cells (Figure 8E) and MHCII^hi^UEA1^+^ cells (Figure 8F) as compared to WT lobes. Remarkably, there were no significant differences between mutant and WT alymphoid FTOCs (Figures 8G,H).

These results conclusively demonstrated that thymocytes were necessary for mTEC maturation but also that the lack of Eph/ephrin-B signaling, that alters thymocyte–TEC interactions, could be affecting RANK/RANKL signaling, mandatory for the proper development of medullary epithelium (14, 38). To test this, we stimulated E14.5 alymphoid FTOCs, isolated from either WT or mutant mice that do not contain mature mTECs, with an agonist anti-RANK antibody for 4 days and observed increased proportions of mature CD40^hi^CD80^+^ cells (Figure 8I) and MHCII^hi^UEA1^+^ cells (Figure 8J) mTECs in both WT and deficient FTOCs, as compared to control ones (Figures 8K,L,O,P). Furthermore, in these experimental conditions RANK stimulation affected WT and EphB-deficient FTOCs similarly (Figures 8M,N). Therefore, these results suggest that the lack of Eph/ephrinB-dependent thymocyte–TEC interactions that occurs in EphB-deficient thymi affects proper mTEC differentiation by altering RANK–RANKL signaling.

## Discussion

Studies on thymic epithelium are essential to understand functional thymocyte differentiation (1, 2) but reported data are controversial and numerous issues remain unresolved. Our current results add new information to this topic demonstrating altered maturation of mTECs, evidenced by the pattern of specific cell marker expression during development, the altered histological organization of medullary epithelial islets in EphB-deficient thymi, particularly in those which lack EphB2, and the phenotype of mutant adult thymus. Eph is a family of protein tyrosine kinase receptors that together with their ligands, ephrins, are involved in thymocyte–TEC interactions and modulate TEC development (20, 22, 23, 25). Furthermore, RANK signaling corrects this defective development. Although we had previously described alterations of the thymic epithelium in EphB-deficient thymi (22), they had never been quantified or related to the molecular machinery known to be involved in the functional maturation of mTECs.

Altered, rather than impaired phenotypic TEC maturation is supported by significant differences between mutant and WT thymi in the expression of different cell markers including those known to be related to functions of the medullary epithelium, such as MHCII and the co-stimulatory molecules, CD40 and CD80 (26, 39). Both WT and mutant developing thymi follow the same pattern of maturation, gradually upregulating expression of the medullary cell marker UEA1, as previously reported (28, 40, 41). However, at both E17.5 and 7PN the proportions of mTECs are significantly lower in mutant, especially in EphB2^−/−^, than those in WT thymi.

Similar conclusions can be drawn from the analysis of other molecules expressed by mTECs that define different cell subsets (39, 42–45). It is assumed that committed mTEPCs differentiate to immature MHCII^lo^ mTECs and then to mature MHCII^hi^ mTECs (26, 46) although mTEC^lo^ cells also include terminally differentiated mTECs (46) as well as osteoprotegerin^+^ and osteoprotegerin^−^ cells (17). Brunk and colleagues (47) also define an MHCII^−^CD80^−^ progenitor cell population capable of upregulating MHCII expression to MHCII^lo^CD80^−^ cells that further generate MHCII^hi^CD80^−^ cTECs and then MHCII^hi^CD80^+^ mTEC.

This upregulation of MHCII expression during TEC development is also evident in both WT and mutant thymi, but slower in the deficient mice especially in EphB2^−/−^ ones that show a more severe phenotype. The expression of both CD40 and CD80 co-stimulatory molecules gradually increases to reach the mature mTEC population that, at E17.5 and 7PN, shows reduced percentages in the mutant thymi, particularly in EphB2^−/−^ mice. CD40 is also expressed in mature cTECs (45) but CD80 expression defines mature mTECs at the end of fetal life and after birth (39). The slow functional maturation of mTEC of EphB-deficient thymi is also supported by the reduced proportions of AIRE^+^ cells observed in both EphB2- and EphB3-deficient thymi, particularly in 7PN EphB2^−/−^ ones. AIRE is a transcriptional factor expressed in a small number of mTECs (48, 49) and involved in the intrathymic presentation of tissue-specific antigens (31), whose lack results in blockade of mTEC maturation and severe autoimmunity in numerous tissues (50, 51).

The condition of mutant adult thymi deserves special comments and confirms the altered maturation of EphB-deficient mTECs. On the one hand, the proportions of adult mTECs, identified by surface markers as UEA1, MHCII, CD40, and CD80, significantly increase, as compared to WT values. However, these results do not presumably reflect a recovery of WT condition in mutant adult thymi, rather, they indicate that defects described during development severely affect the adult thymic cortex reducing its volume and, accordingly, the proportions of cTECs with the consequent increased percentage of mTECs. We previously reported this condition immunohistochemically (22) and more recently quantified it by flow cytometry analysis (25). Remarkably, this cortical phenotype is particularly severe in EphB3-deficient mice (25) and, thus, the proportions of adult mTECs are significantly higher in these mutants than in EphB2^−/−^ thymi. By contrast, the proportions of AIRE^+^ cells in mutant adult thymi remain significantly lower than in WT thymi, presumably reflecting defects in RANK/RANKL signaling necessary for full maturation of mTECs (2). On the other hand, these results indicate that the found phenotype in mutant embryonic thymus is not recovered in adult mice, but rather becomes more severe as a consequence of the gradual accumulation of defects. Therefore, we might conclude that the lack of EphB2 or EphB3 results in an altered program of TEC development, as compared to that of WT cells.

Alterations in the development of distinct TEC populations also occur in mutant Cld3,4^+^ mTEPCs. Cld3,4^+^ cells appear in the early thymic primordium as committed precursor cells of mTECs (32, 52). A Cld3,4^+^ cell subset that expresses MTS20 includes immature TECs whereas Cld3,4^hi^SSEA1^+^ cells capable of self-renewal, generate mTECs and are also considered to be committed medullary precursor cells (9).

Recently, we reported delayed maturation of the total immature MTS20^+^ TECs in both embryonic EphB2- and EphB3-deficient thymi (24). Our current results indicate that at E15.5 when Cld3,4^+^MTS20^+^ cells disappear from WT thymi, they still remain in both EphB2^−/−^ and EphB3^−/−^ ones. Likewise, the proportions of Cld3,4^hi^SSEA1^+^ cells that gradually decrease along thymus development (9) remain significantly high in mutant thymi. It has been reported that embryonic and postnatal β5t progenitors, whose percentages are also reduced in EphB-deficient thymi (25) give rise to Cld3,4^hi^SSEA1^+^ cells (8), that appear earlier in ontogeny than RANK^+^ mTECs presumably involved in the further maturation of medullary epithelium (7, 53). On the other hand, in postnatal WT thymi, when medullary areas increase (28), the proportions of total Cld3,4^hi^ cells are significantly higher than in both EphB2^−/−^ and EphB3^−/−^ ones. Taken together, these data conclusively indicate that EphB, especially EphB2, are involved in the maturation of medullary thymic epithelium, affecting both immature and mature mTECs but also progenitor cells committed to the mTEC lineage.

The lack of EphB signaling affects also the thymic histological organization because WT RTOCs supplied with inhibitory anti-EphB2 or anti-EphB3 antibodies exhibit a similar morphology showing more and smaller K5^+^ medullary areas than control ones. In addition, RTOCs established with EphB-deficient thymic lobes contain significantly higher numbers of small medullary islets than WT re-aggregates. Presumably, the lower numbers of mTEC present in mutant thymi could explain the reduced expansion of medullary areas whereas, around birth, in WT thymus, medullary islets expand and fuse to constitute a unique central medulla (19, 28).

In order to evaluate the role played by TECs and thymocytes in the phenotype of mutant mTECs, an issue that does not allow the analysis of knockout mice, we grafted for 3 weeks E13.5 mutant alymphoid FTOCs under the kidney capsule of WT mice as well as WT alymphoid FTOCs in mutant mice. In any combination, the presence of mutant TECs or thymocytes results in reduced cellularity and a phenotype quite similar to that observed in adult thymi, as corresponding to FTOCs maintained *in vivo* growing for three weeks, with significant decreased proportions of Ly51^+^UEA1^−^ cTECs and increased values of Ly51^−^UEA1^+^ mTECs. These results confirm previous ones demonstrating a role of TEC in the thymic phenotype of EphB-deficient mice (54), but also a non-autonomous contribution of thymocytes (55).

On the other hand, although both molecules, EphB2 and EphB3, are necessaries for a proper epithelial maturation, the lack of EphB2 produces a more severe medulla phenotype, whereas the absence of EphB3 seems to particularly affect thymic cortex development (25). In supporting these results, we had previously demonstrated that E13.5 alymphoid fetal thymus lobes derived from EphB2^−/−^ mice grafted for 4 weeks under the kidney capsule of WT mice show a more fragmented medulla than those derived from EphB3^−/−^ thymi (54). Other studies have suggested that the role of EphB3 signaling in other processes occurring in the thymus, including T-cell differentiation (55) or LP cell seeding (56, 57), is less important than those mediated through EphB2. All these results demonstrate a specificity of the EphB-mediated responses ignored by other authors (58–60).

Moreover, despite the reported changes in EphB-deficient thymi (22, 61) and the altered maturation of thymic medulla demonstrated herein, a thymic compartment key for generation of central tolerance, these mice live in non-sterile conditions and do not show apparent immunological deficits (20, 62). Other mice deficient in several Eph have no phenotype (59, 60), and in other experimental models, altered mature mTEC do not course with autoimmune reactivity (63). Other authors however, have concluded that ephrin-B1 and ephrin-B2, the main ligands of EphB2 and EphB3, are necessary for the proper development of Th1 and Th2 cell subsets (64–66). Some authors have explained these results indicating that defects in some thymic areas do not affect the functional maturation of thymocytes (67, 68). In agreement, Cosway et al. (38) recently reported that profound perturbations of mTEC caused by specific deletion of LTβR gene in TEC do not result in altered tolerance. In our model, morphological changes occurring in EphB-deficient thymi course with altered appearance and maturation, rather than total disappearance, of mTEC markers that could be sufficient for the generation of functional T cells, although this assumption requires further confirmation.

On the other hand, we analyzed possible molecules involved in the altered maturation of mTEC in mutant thymi. In this respect, although CCL19 transcripts, that together with CCL21, attracts positively selected CCR7^+^ thymocytes to the adult thymic medulla (69, 70), are significantly reduced in E15.5 fetal EphB-deficient thymi, presumably this feature does not affect mTEC development, because at E15.5 the thymic medulla is not defined and there are no positively selected thymocytes. The reduced expression of these chemokines in fetal EphB-deficient thymi is rather associated with defects in thymocyte migration throughout the thymic parenchyma (57). Furthermore, there are no changes either in the transcript production of LTβR and RANK in both fetal and PN mutant thymi, two receptors of the TNF receptor superfamily expressed on mTEC and whose involvement in TEC development is clearly recognized although not conclusively understood. LTβR signaling in mTEC seems to control the population size of mTEPCs (46) and influences mTEC development and organization (43, 46, 71), but its claimed relationship with the Fezf2 transcription factor (72) is questioned by other authors (38). Meantime, RANK and CD40 signaling are important for AIRE mTEC development (2) and, also perhaps for Fezf2 regulation (38). Indeed, RANKL stimulate RANK signaling promoting maturation of immature CD80^−^AIRE^−^ cells to mature CD80^+^AIRE^+^ mTECs (14).

Although there are no changes in the levels of RANK transcripts in EphB-deficient thymi, alterations in the lymphoid cells that express RANKL and/or a partial disruption of cell-to-cell interactions necessary for RANK signaling could also explain the altered maturation of mutant thymic medulla. Our current results suggest that both situations occur in EphB-deficient thymi. In adult thymus, RANK signaling is mediated by RANKL-expressing positively selected CD4^+^ thymocytes (36, 73) but during embryonic development, when there are no SP thymocytes, RORγt^+^ lymphoid tissue-inducing cells (26) and canonical Vγ5^+^ T cells (37) are involved. Our current results show decreased proportions of Vγ5^+^RANKL^+^ cells in E15.5 mutant thymi, as previously observed in total γδ thymocytes (74) that could consequently reduce inductive signals in RANK-expressing mTEC. Furthermore, a lower incidence of cell-to-cell contacts in the mutant thymic lobes that would result in a lower RANK signaling and, consequently, in reduced mTEC maturation is suggested indirectly by the histological organization of EphB-deficient thymic epithelium. In both EphB2^−/−^ and EphB3^−/−^ thymi, there are profound changes in the epithelial cell morphology that shows reduced and shortened cell processes (22), increased proportions of apoptotic TEC (75) and large areas devoid of TECs (23). In addition, lower numbers of LP cells seed the fetal mutant thymi (24, 57). All these findings obviously contribute to disruption of the TEC network and the thymocyte–TEC contacts.

We then tested whether direct RANK signaling by providing anti-RANK antibodies rescued mTEC maturation in alymphoid FTOCs. We first confirmed, as previously reported (14), the involvement of thymocytes in mTEC maturation because in their absence there are no mature CD40^hi^CD80^+^ cells or MHCII^hi^UEA1^+^ mTECs either in WT or mutant FTOCs. Furthermore, defective mTEC maturation is recovered by supplying agonist anti-RANK antibodies for 4 days to both WT and mutant alymphoid FTOCs, demonstrating that the RANK stimulation mediated by RANKL-expressing cells can be substituted by direct stimulation of the receptor in the presence of reduced lymphoid cells. These results indicate that, in some way, the reduced EphB-mediated thymocyte–TEC interactions in EphB-deficient thymi, rather than the own lack of EphB, affect the molecular machinery, including RANK signaling, which controls mTEC maturation.

## Data Availability Statement

The raw data supporting the conclusions of this manuscript will be made available by the authors, without undue reservations, to any qualified researcher.

## Ethics Statement

The study was carried out in accordance with the recommendations of the “Ethic Committee for Animal Research” of Complutense University. The protocols were approved by the Regional Government of Madrid.

## Author Contributions

SM-H and JG-C performed experiments and analyzed the data. AZ designed the experiments, analyzed the data, and wrote the manuscript.

## Conflict of Interest Statement

The authors declare that the research was conducted in the absence of any commercial or financial relationships that could be construed as a potential conflict of interest.
