# Supplementary material for: Altered Maturation of Medullary TEC in EphB-Deficient Thymi Is Recovered by RANK Signaling Stimulation
Source: Front Immunol. 2018 May 9;9:1020. doi: 10.3389/fimmu.2018.01020 (PMC5954084; doi:10.3389/fimmu.2018.01020)
Supplement: Supplementary file 1 [file Image_1.PDF]

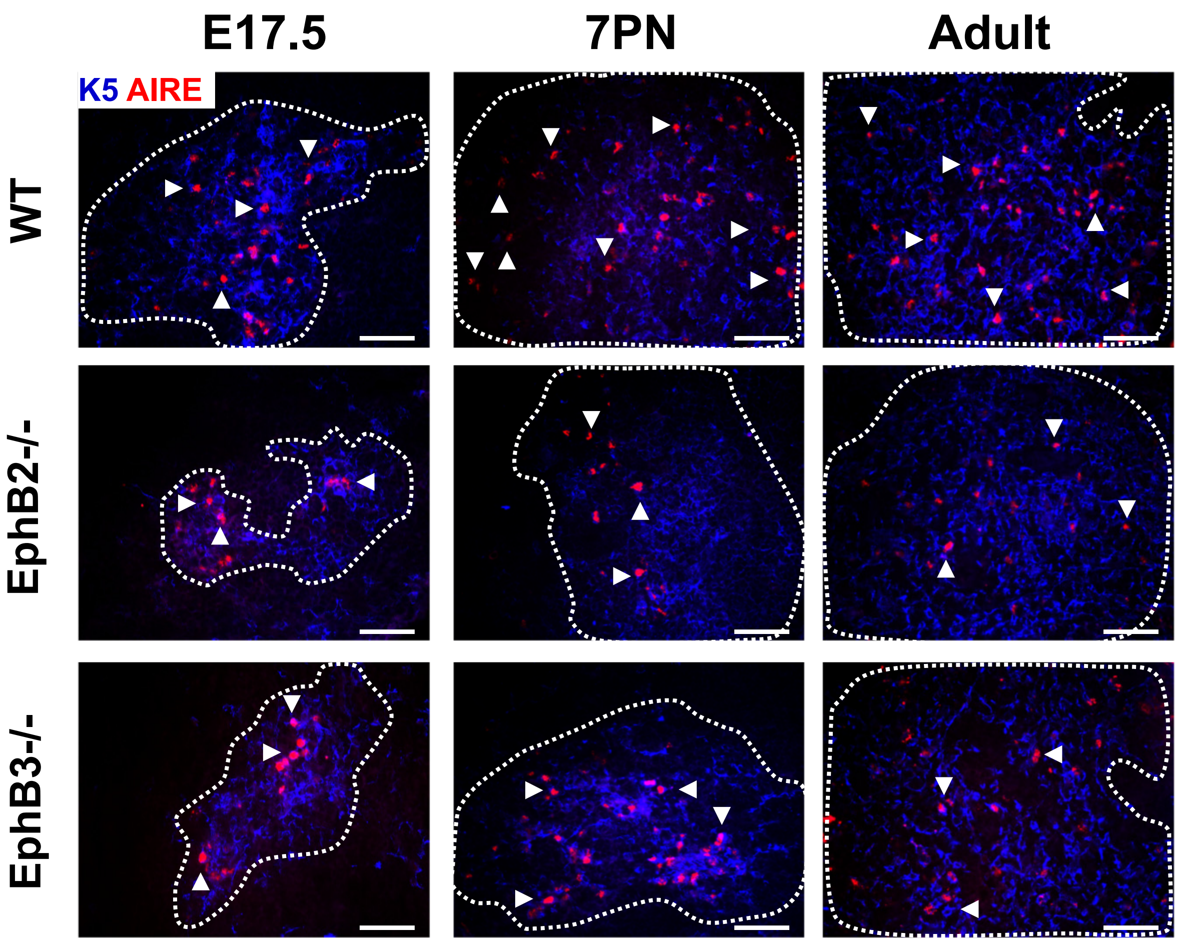

Supplementary Figure 1. Presence of AIRE⁺ cells in E17.5, 7PN and adult WT and EphB-deficient thymi. AIRE⁺ cells (arrowheads) are present in K5⁺ medulla (dotted line) in both WT and EphB-deficient mice. Scale: 50µm
