# Supplementary material for: Altered Maturation of Medullary TEC in EphB-Deficient Thymi Is Recovered by RANK Signaling Stimulation
Source: Front Immunol. 2018 May 9;9:1020. doi: 10.3389/fimmu.2018.01020 (PMC5954084; doi:10.3389/fimmu.2018.01020)
Supplement: Supplementary file 2 [file Image_2.PDF]

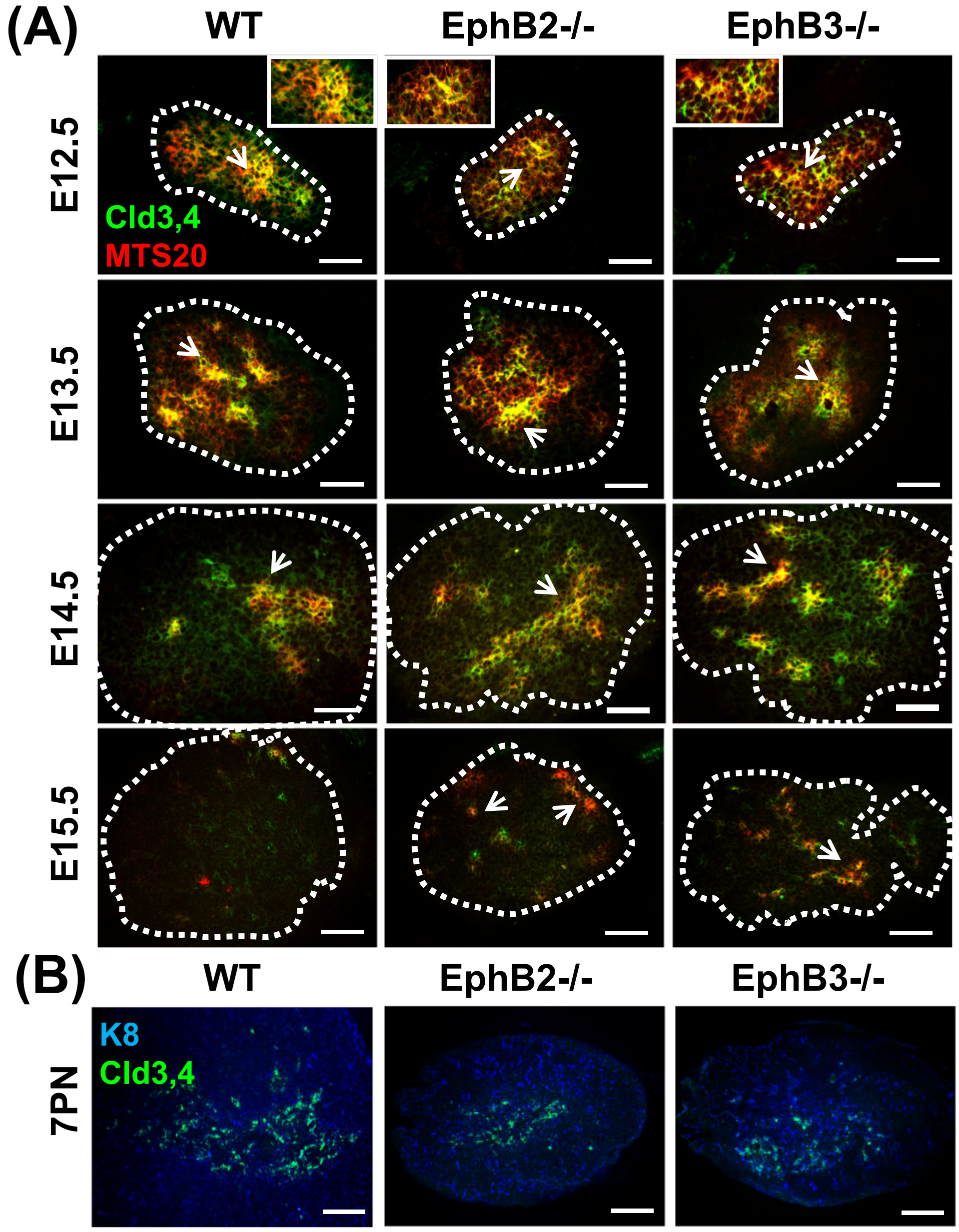

Supplementary Figure 2. Cld3,4^{+} cells in both fetal and postnatal WT and EphB-deficient thymi. **(A)** Cld3,4^{hi}MTS20^{hi} cells (arrows) decrease throughout development (E12.5-E15.5) of WT thymi whereas in mutant ones they remain from E14.5 onward. Scale bar: 50µm (E12.5-E14.5) and 100µm (E15.5). **(B)** Expression of Cld3,4^{hi} cells (green) throughout the K8^{+} thymus parenchyma (blue) of 7PN WT and mutant thymi. Scale: 200µm.
