# Supplementary material for: Altered Maturation of Medullary TEC in EphB-Deficient Thymi Is Recovered by RANK Signaling Stimulation
Source: Front Immunol. 2018 May 9;9:1020. doi: 10.3389/fimmu.2018.01020 (PMC5954084; doi:10.3389/fimmu.2018.01020)
Supplement: Supplementary file 3 [file Image_3.PDF]

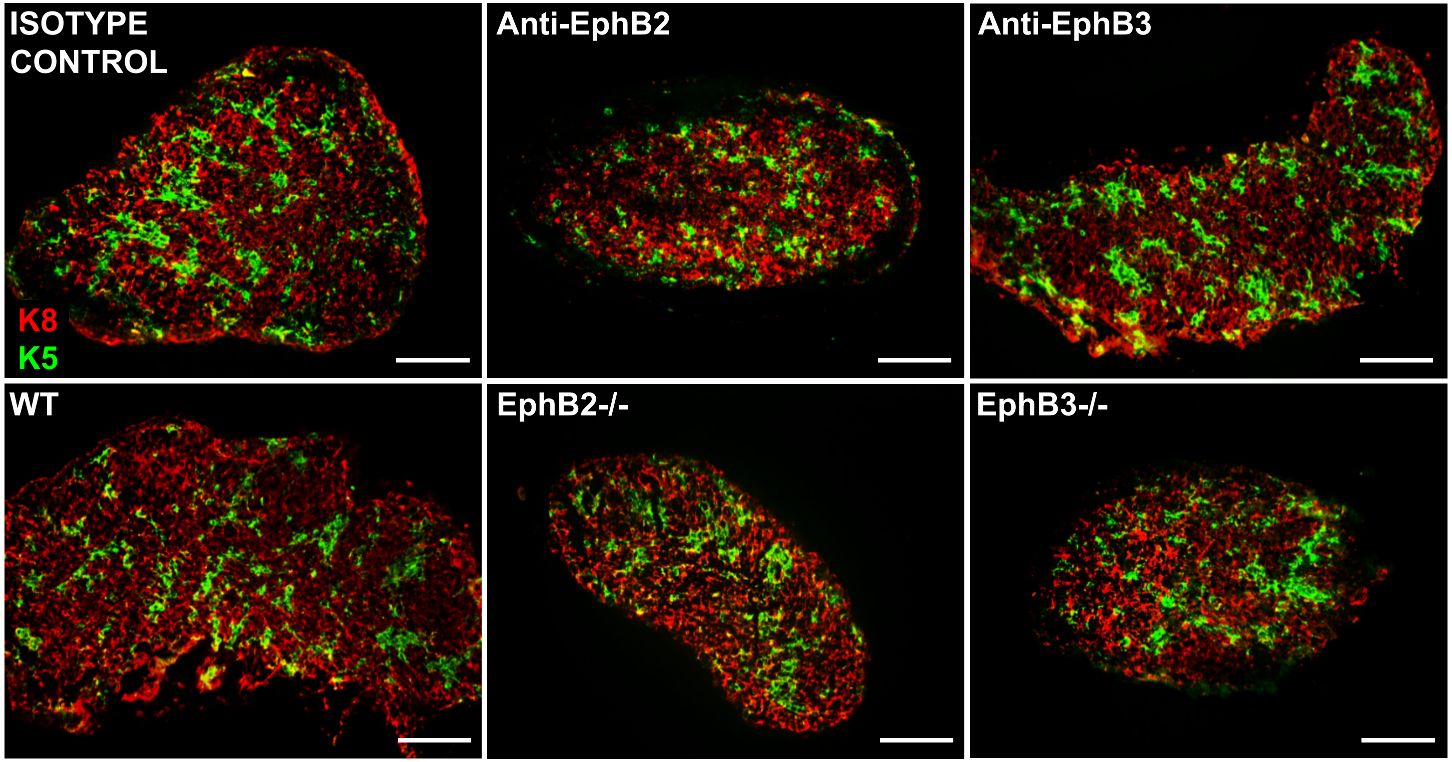

Supplementary Figure 3. $K5^+$ medullary areas (green), identified using an anti-K5 antibody, in either WT RTOCs treated with either anti-EphB2 or anti-EphB3 antibodies or established with EphB-deficient cells. Both WT RTOCs treated with anti-EphB2 or anti-EphB3 antibodies and mutant RTOCs contain higher numbers of $K5^+$ medullary areas (green) but smaller than their respective controls. A minimum of five non-overlapping sections of at least three different RTOCs was analyzed. Scale: 100µm.
